# Reference-Based Layer Echo Suppression for Ultrasonic Evaluation of Thick Pultruded CFRP Composites

**DOI:** 10.3390/ma19214566

**Published:** 2026-10-29

**Authors:** Hun-Hee Kim, Bong-Ju Kim, Yun-Taek Yeom

**Affiliations:** 1 Doosan Enerbility, Changwon-si 51711, Republic of Korea; 2 Department of Smart Mechanical Engineering, Dongyang University, Yeongju-si 36040, Republic of Korea; kbj1952@dyu.ac.kr

**Keywords:** pultruded CFRP, ultrasonic nondestructive evaluation, layer echo suppression, reference-based signal processing, wavelet denoising, hilbert-envelope analysis

## Abstract

Pultruded carbon-fiber-reinforced polymer (CFRP) composites are increasingly used in large wind turbine blades, but ultrasonic evaluation of thick pultruded structures is complicated by repetitive layer reflections and prolonged transducer ringing. This study proposes a reference-based Layer Echo Suppression Method (LESM) to suppress deterministic structural responses that can mask internal defect echoes, thereby improving the visibility and identification of damage-related reflectors in thick CFRP components. Ultrasonic A-scan signals were acquired from sound and defect regions of a 100-mm-thick pultruded CFRP specimen containing an artificial defect at approximately 50 mm using a 0.5 MHz single-element contact transducer. LESM combines cross-correlation-based registration, independent wavelet denoising, reference-based Hilbert-envelope differencing, and positive-residual weighting of the denoised defect signal. The normalized cross-correlation coefficient between the reference and defect signals was 0.9941. DBR-based evaluation selected Sym5 at decomposition level 5. The final LESM signal yielded a defect-to-background ratio (DBR) of 34.82 dB, compared with 14.14 dB for the raw signal, an improvement of 20.68 dB. The defect-related waveform near 50 mm was retained while surrounding layer echoes and ringing were substantially reduced. By enhancing localized defect reflections without requiring additional ultrasonic hardware, LESM can support the identification of internal planar damage-like discontinuities in thick CFRP components using conventional pulse-echo measurements. These results demonstrate the proof-of-concept feasibility of the proposed method under the examined condition.

## 1. Introduction

The rapid expansion of renewable energy has accelerated the deployment of large-scale wind turbines as an effective means of reducing carbon emissions and mitigating climate change. To improve power-generation efficiency, wind turbine blades have continuously increased in size, resulting in growing demand for lightweight materials with high stiffness, strength, and fatigue resistance [1,2,3,4,5]. Both glass-fiber-reinforced polymer (GFRP) and CFRP composites are widely used in wind turbine blade structures. GFRP offers relatively low material cost, good corrosion resistance, and adequate strength and fatigue performance, making it suitable for large-volume blade components such as skins and shear webs. In contrast, CFRP provides higher specific stiffness and strength as well as favorable fatigue resistance, although at a higher material cost, and is therefore particularly advantageous for highly loaded components such as spar caps. The long-term durability of both material systems depends on environmental exposure and fiber–matrix interfacial stability. GFRP may undergo degradation under moisture and alkaline conditions, whereas the mechanical performance of CFRP can also evolve under hygrothermal and freeze–thaw environments. Carbon–glass hybrid configurations have consequently been investigated to combine the cost advantages of glass fibers with the mechanical and environmental resistance provided by carbon fibers [6,7]. Carbon-fiber-reinforced polymer (CFRP) composites have therefore been increasingly incorporated into load-carrying components of large wind turbine blades because of their high specific stiffness and potential for structural weight reduction [1,2].

Among the manufacturing technologies used for CFRP blade components, pultrusion has attracted considerable attention for the fabrication of spar-cap elements in large-scale wind turbine blades [8,9,10,11]. Pultrusion enables the continuous production of unidirectional CFRP components with high fiber alignment and manufacturing repeatability, making it particularly suitable for thick load-carrying structures [8,9,10,11]. However, the resulting material contains periodically distributed fiber–resin interfaces and resin-rich regions throughout its thickness. From an ultrasonic inspection perspective, these internal interfaces generate repetitive structural reflections because of local acoustic-impedance variations. The interpretation of pulse-echo signals is further complicated in thick components, where low-frequency ultrasonic excitation is required to achieve sufficient penetration. The prolonged temporal response of a low-frequency transducer can cause adjacent layer echoes and ringing components to overlap, thereby increasing the ultrasonic background level around a defect response.

**Figure 1:**
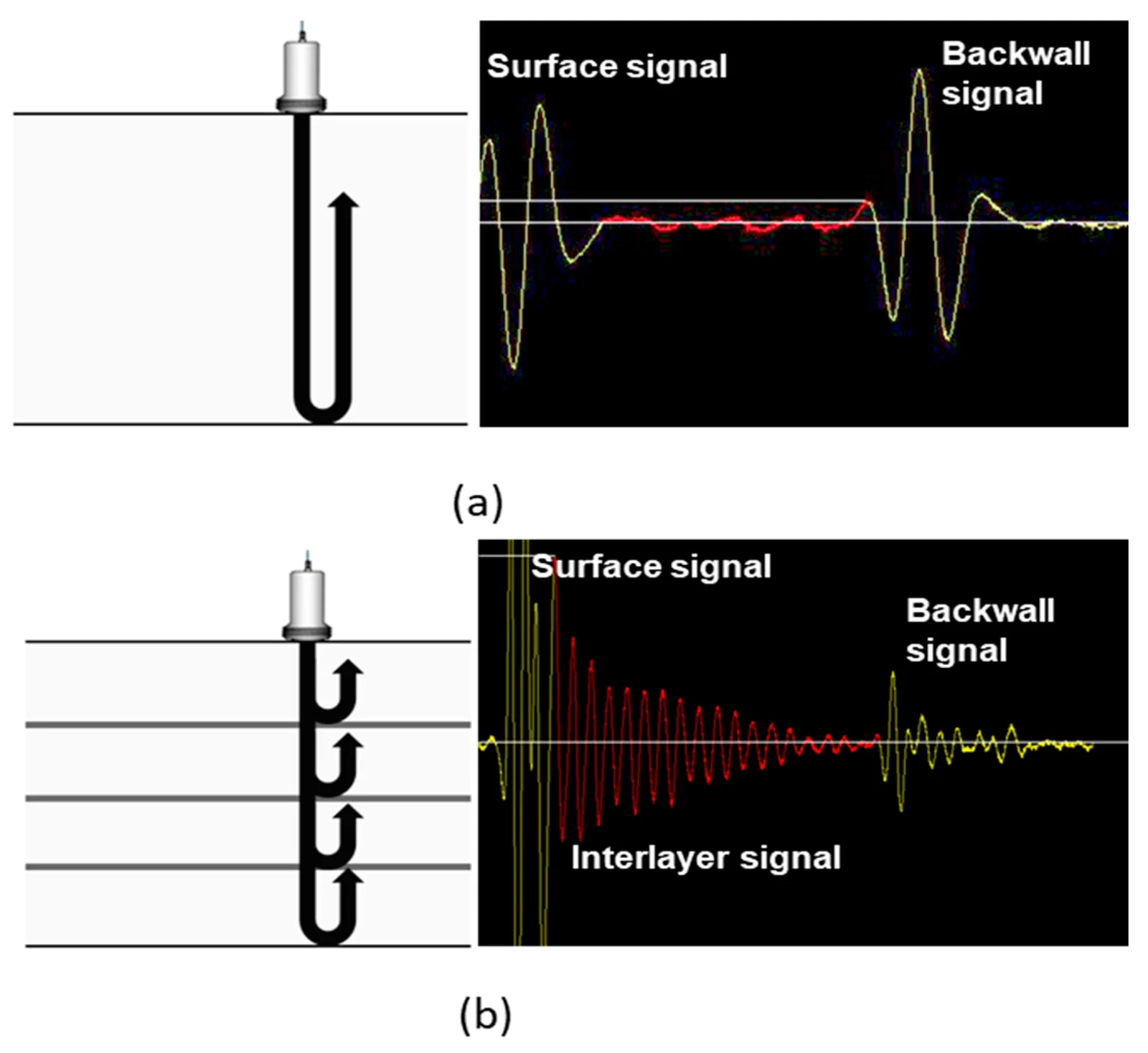
Schematic comparison of ultrasonic pulse-echo responses in CFRP composites: (**a**) prepreg and (**b**) pultruded CFRP.

Figure 1 schematically compares the ultrasonic pulse-echo responses of conventional prepreg and pultruded CFRP composites. In prepreg CFRP, the dominant ultrasonic responses are generally associated with the front surface, internal discontinuities, and back wall, although structural noise can also be generated by the laminate architecture. In thick pultruded CFRP, the periodically repeated fiber–resin structure produces additional intermediate echoes throughout the inspection depth. These deterministic structural responses can overlap with defect-related reflections and reduce the contrast between a defect echo and its surrounding background.

Various nondestructive testing techniques have been investigated for evaluating wind turbine blades and CFRP composite structures. Contact pulse-echo ultrasonics and guided-wave techniques have been applied to wind turbine blade inspection [12,13], while thermographic approaches have also been investigated for blade condition monitoring [14]. For CFRP components, phased-array ultrasonic testing and total focusing methods have been employed to characterize material properties and improve imaging performance in anisotropic laminates [15,16]. More recently, automated ultrasonic inspection and laser-ultrasonic techniques have been investigated to improve inspection efficiency and defect characterization in complex CFRP structures [17,18,19]. These studies demonstrate the applicability of ultrasonic NDE to composite structures; however, reliable interpretation remains challenging when defect-related echoes coexist with deterministic structural responses generated by the composite architecture.

A range of signal-processing approaches has therefore been introduced to improve defect-to-background separation in ultrasonic measurements. Benammar et al. applied split-spectrum processing and an expectation-maximization algorithm to improve the detection of delamination defects in CFRP laminates [20]. Wang et al. investigated signal-processing approaches for separating near-surface flaw responses from strong interface signals in CFRP [21]. Hybrid approaches combining cross-correlation, wavelet analysis, and Hilbert-based processing have also been proposed for ultrasonic defect estimation in composite structures [22]. In addition, signal-processing algorithms based on non-contact-excited Lamb waves [23] and Hilbert-transform processing combined with improved variational mode decomposition [24] have been developed for delamination detection. Recent studies have further addressed material-induced artifacts by decoupling propagation characteristics from texture-induced responses in woven CFRP [25] and by reconstructing defect features from ultrasonic scan data using local-outlier-based processing [26].

The suppression and compensation of reverberation-related ultrasonic responses have also been studied in other inspection and imaging configurations. Claesson and Salomonsson proposed frequency- and depth-dependent compensation to account for depth-dependent signal distortion that cannot be adequately corrected using conventional time-gain compensation [27]. Shin et al. developed multi-phase apodization with cross-correlation to suppress reverberation clutter and improve image contrast in array-based medical ultrasound imaging [28]. Han et al. investigated ultrasonic imaging through reverberating media and demonstrated the suppression of structural noise, including surface and reverberation waves, in thin-layer configurations [29]. Chen et al. proposed a phase-circular-statistics-vector method to suppress secondary interface echoes in immersion ultrasonic phased-array imaging [30]. More recently, Schäfer et al. investigated echo cancellation for flaw detection behind reverberating layers and compared the approach with background subtraction and time-gain compensation [31]. Collectively, these studies demonstrate that deterministic reverberation and interface responses should be addressed differently from random measurement noise.

Nevertheless, the inspection configuration considered in the present study differs from those addressed in much of the previous literature. Existing reverberation-suppression methods have primarily focused on array-based imaging, medical ultrasound, thin reverberating layers, secondary immersion-interface echoes, or flaws located behind a discrete reverberating layer [27,28,29,30,31]. In a thick pultruded CFRP component, repetitive echoes are generated throughout the material thickness by the intrinsic fiber–resin architecture, and industrial inspection may be performed using a low-frequency single-element contact transducer. Under these conditions, the structural responses are present directly in the A-scan signal and can exhibit similar waveform characteristics in adjacent sound and defect-containing regions. This repeatability provides an opportunity to use a signal acquired from a sound region as a structural reference for suppressing common layer-echo components.

The specific methodological contribution of LESM lies in this reference-derived envelope-residual weighting architecture rather than in the individual use of cross-correlation, wavelet denoising, or Hilbert-envelope analysis. The proposed architecture differs from direct reference subtraction in three respects: it uses a sound-region signal to characterize deterministic layer echoes, derives a non-negative weighting function from localized envelope differences rather than using a signed waveform residual as the final signal, and applies this weighting function to the denoised defect waveform to preserve its oscillatory characteristics. The method was experimentally examined using pulse-echo A-scan signals acquired from a 100-mm-thick pultruded CFRP specimen containing an artificial delamination reflector at a depth of approximately 50 mm. Because the present experiment considers a single artificial reflector under a uniform-thickness condition, the results are presented as a proof-of-concept evaluation rather than as a comprehensive validation across different defect geometries and inspection conditions.

## 2. Specimen and Methods

### 2.1. Experimental Setup

#### 2.1.1. Pultruded CFRP Specimen

A pultruded carbon fiber reinforced polymer (CFRP) composite representative of the spar-cap structure used in large-scale wind turbine blades was employed in this study. Unlike conventional prepreg CFRP composites, which are fabricated by manually stacking prepreg sheets followed by curing, pultruded CFRP composites are continuously manufactured by impregnating unidirectional carbon fibers with thermosetting resin and curing them through a heated die. According to construction information provided by the manufacturer, the specimen was assembled from ten pultruded CFRP layers bonded with resin, corresponding to a nominal thickness of approximately 10 mm per layer. Thin resin-rich interfaces were formed between adjacent layers. These interfaces generate repetitive ultrasonic reflections owing to the acoustic impedance mismatch between the carbon fiber layers and the resin-rich regions, making ultrasonic signal interpretation considerably more challenging than that of conventional prepreg composites. The fabricated pultruded CFRP specimen had dimensions of 2000 mm in length, 400 mm in width, and 100 mm in thickness, corresponding to a representative section of a wind turbine blade spar cap. The pultrusion direction and predominant carbon-fiber direction coincided with the 2000-mm longitudinal direction of the specimen. Ultrasonic measurements were performed from the broad surface of the specimen, such that the primary propagation direction was through the 100-mm thickness and approximately perpendicular to the predominant fiber direction. The reference and defect measurement regions had the same nominal thickness and material architecture.

**Figure 2:**
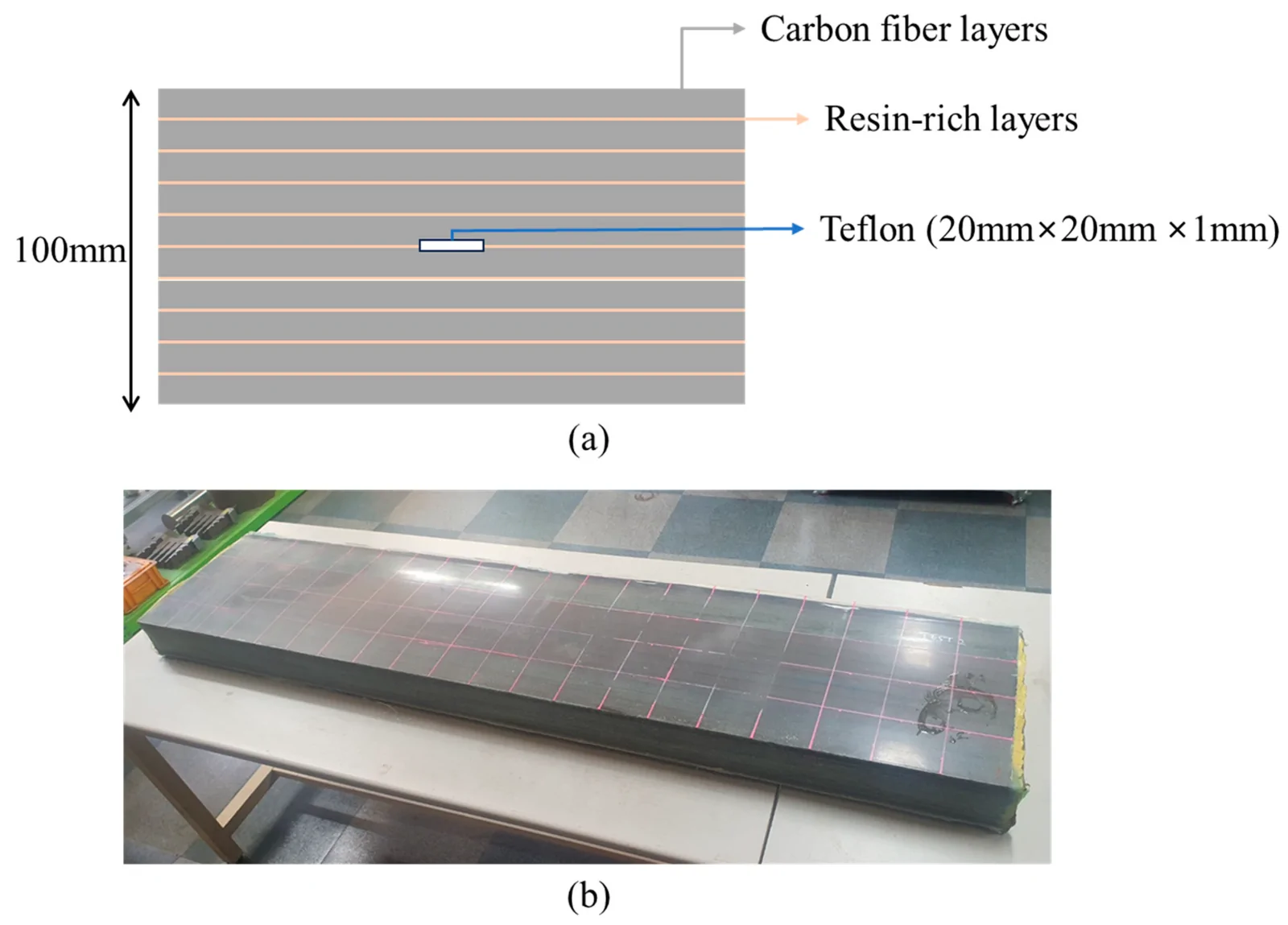
Pultruded CFRP Specimen: (**a**) schematic cross-section and (**b**) fabricated specimen.

**Figure 3:**
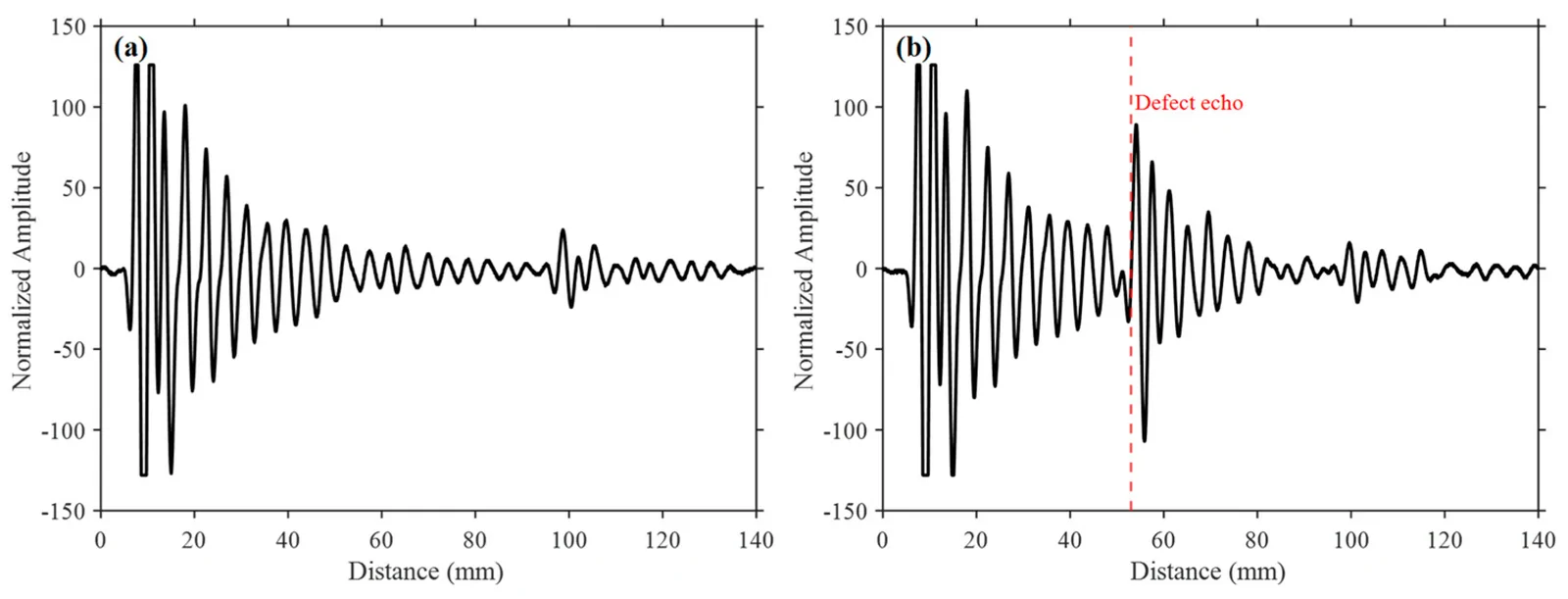
A-scan signals of the pultruded CFRP specimen: (**a**) reference signal (sound region) and (**b**) defect signal.

To simulate an internal planar delamination, a 20 mm × 20 mm × 1 mm polytetrafluoroethylene (PTFE, commonly referred to as Teflon) insert was embedded during manufacturing at the mid-thickness of the specimen, corresponding to a depth of 50 mm from the ultrasonic inspection surface. Teflon is commonly used as an artificial delamination reflector in ultrasonic inspection because of its low acoustic impedance and weak interfacial bonding with the surrounding composite material, thereby providing a repeatable and well-defined defect response [18,32]. Figure 2 presents the pultruded CFRP specimen used in this study. Figure 2a, which is schematic and not drawn to scale, illustrates the ten pultruded CFRP layers, thin resin-rich interfaces, and the embedded Teflon insert. Figure 2b shows the fabricated pultruded CFRP specimen. The periodically repeated fiber-resin interfaces constitute the primary source of the interlayer echoes investigated in this study, while the embedded Teflon insert provides a representative artificial defect for evaluating the proposed Layer Echo Suppression Method (LESM). At the 0.5 MHz center frequency, reflections from adjacent interfaces overlap with the long-duration transducer response; therefore, the oscillations in Figure 3a represent superposed interface echoes and ringing rather than individually resolved layer echoes.

#### 2.1.2. Ultrasonic Experimental Setup

Ultrasonic pulse-echo measurements were performed using an EPOCH XT ultrasonic flaw detector (Olympus, Tokyo, Japan) equipped with a 0.5 MHz single-element contact transducer (Panametrics M2008) with a diameter of 1 inch. A gel-type ultrasonic couplant was applied between the transducer and the specimen surface to ensure stable acoustic coupling during signal acquisition. All measurements were performed manually without an automated scanning system. The transducer was directly coupled to the specimen surface without a wedge and maintained at an approximately normal incidence, with the inspection configuration intended to generate a predominantly through-thickness quasi-longitudinal response. The relatively low center frequency was selected to provide sufficient ultrasonic penetration through the approximately 100-mm-thick specimen while maintaining stable signal acquisition throughout the thickness.

All A-scan waveforms were acquired using identical pulser–receiver settings and exported as signed RF amplitude data without rectification. The material sound velocity used for depth calibration was set to 2500 m/s. No acquisition filter was applied, and five waveform averages were used for each stored A-scan. Each exported waveform consisted of 13,000 uniformly spaced samples represented over a calibrated depth range of 0–140 mm, corresponding to a depth increment of approximately 0.01077 mm per sample. The receiver gain was adjusted before data acquisition to provide clear visualization of the through-thickness ultrasonic responses and was then maintained unchanged for the sound-region reference signal and both defect-region measurements. Although the numerical gain setting was not retained in the experimental record, no gain readjustment was performed between these measurements. No additional resampling was applied before LESM processing.

The reference and defect signals used in the LESM analysis were single-point A-scans acquired at manually selected locations rather than over a spatial grid. The defect-region A-scan was acquired from the inspection surface over the projected area of the embedded PTFE insert. The reference A-scan was acquired from a nearby sound region on the same inspection surface, laterally outside the projected PTFE-insert boundary. Both measurement locations were within the same locally uniform section of the specimen and had the same nominal thickness of 100 mm and the same material architecture. The transducer was maintained at approximately normal incidence at both locations. Therefore, the principal difference between the two measurement locations was the presence or absence of the embedded PTFE insert. The high normalized cross-correlation coefficient of 0.9941 confirmed the close correspondence of the structural echo patterns acquired at the two locations. To minimize measurement-related differences, the reference and defect signals were acquired using the same transducer, pulse-echo settings, and coupling procedure. Any remaining temporal mismatch caused by manual probe positioning or local coupling variations was subsequently evaluated using cross-correlation-based signal registration and corrected when necessary.

The ultrasonic A-scan signals acquired from the specimen were subsequently used as the input data for the proposed Layer Echo Suppression Method (LESM). Figure 3 presents representative ultrasonic signals obtained from the pultruded CFRP specimen, where Figure 3a shows the A-scan signal acquired from the sound reference region and Figure 3b shows the A-scan signal acquired from the defect region.

To provide a limited assessment of measurement repeatability, two defect-region A-scans were acquired at the same nominal defect location through separate manual contact measurements. After the first acquisition, the transducer was completely removed from the specimen surface and then manually repositioned at the same nominal location under an approximately normal-incidence condition. The same transducer, instrument settings, and coupling procedure were used for both acquisitions. The first acquisition, which is presented in Figure 3b, was used for method development and parameter selection. The second acquisition was processed using the same sound-region reference signal and the fixed LESM parameters selected from the first acquisition, without further parameter optimization.

Because of the relatively low center frequency and the resulting long-duration ultrasonic response, pronounced ringing is observed in the A-scan signals. Consequently, repetitive layer echoes coexist with the defect reflection and increase the surrounding background level. As shown in Figure 3b, the defect reflection at approximately 50 mm is identifiable in the raw A-scan but is accompanied by strong layer echoes and prolonged ringing. Therefore, the objective of the proposed LESM is to suppress these common structural responses and quantitatively improve defect-to-background separation while preserving the defect-related waveform.

### 2.2. Layer Echo Suppression Method (LESM)

**Figure 4:**
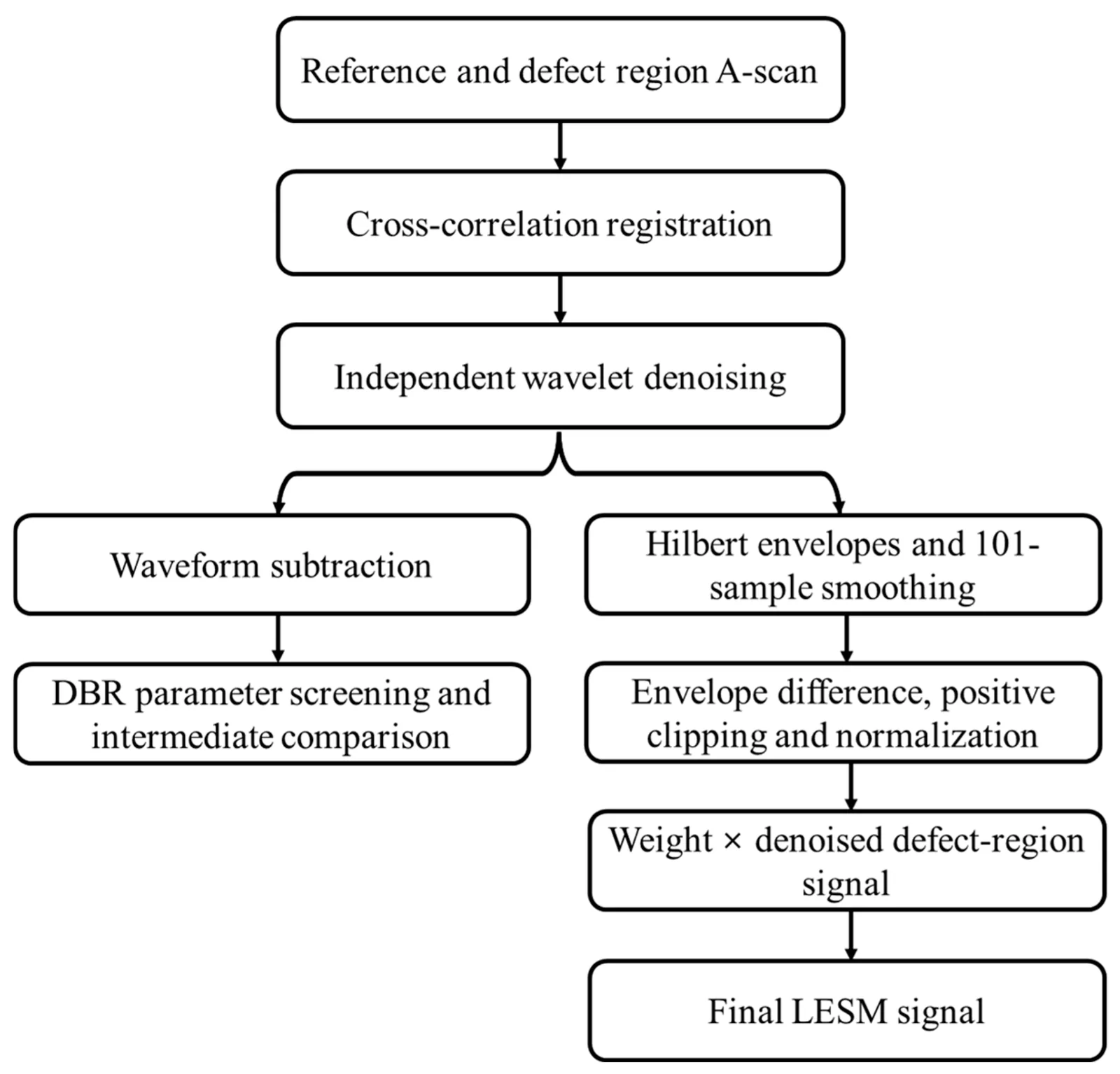
Schematic diagram of the proposed Layer Echo Suppression Method (LESM).

The proposed Layer Echo Suppression Method (LESM) uses an ultrasonic A-scan acquired from a sound region as a structural reference for suppressing repetitive responses common to the reference and defect regions. The main LESM processing path comprises (i) cross-correlation-based registration, (ii) independent wavelet denoising of the aligned reference and defect signals, (iii) Hilbert-envelope extraction and moving-average smoothing, (iv) formation and normalization of the positive envelope residual, and (v) pointwise weighting of the wavelet-denoised defect signal. Waveform-level reference subtraction is additionally used as an auxiliary analysis for wavelet-parameter evaluation and intermediate comparison; it is not used as the direct input to the final LESM weighting stage. The overall processing flow is illustrated in Figure 4.

#### 2.2.1. Cross-Correlation-Based Signal Alignment

The reference and defect A-scans may exhibit a small temporal mismatch because of manual probe positioning, coupling variations, and local differences in ultrasonic propagation. Even a small mismatch can produce artificial residual components when the signals are compared. Therefore, the temporal correspondence between the two signals was evaluated before the subsequent processing stages.

The mean value was removed from each complete A-scan before the registration gates were extracted. The registration interval was defined as 27–48 mm, which is located before the nominal defect response near 50 mm. This interval excludes the strong front-surface response and the defect-related reflection while retaining representative repetitive layer echoes. Let *x_r_*(*n*) and *x_d_*(*n*) denote the mean-removed reference and defect signals within the registration interval, respectively. Their normalized discrete cross-correlation was calculated as

(1) $$R_{\mathrm{rd}}\left(\tau \right)=\frac{\sum_{n=0}^{N-1}x_{r}(n)x_{d}(n+\tau )}{\sqrt{\sum_{n=0}^{N-1}x_{r}^{2}(n)}\sqrt{\sum_{n=0}^{N-1}x_{d}^{2}(n+\tau )}}$$

where *τ* is the lag in samples. The cross-correlation was evaluated over a maximum lag of ±500 samples using the coefficient-normalized form. Consistent with the implemented MATLAB 2024b procedure, the optimal integer lag was selected from the maximum value of the normalized cross-correlation, rather than from the maximum absolute value:

*τ*^∗^ = arg max *R_rd_*(*τ*) (2)

The reference signal was then shifted by *τ*^∗^ samples and zero-filled at the vacated boundary. A positive lag shifted the reference signal toward increasing sample number, whereas a negative lag shifted it toward decreasing sample number. The defect signal was not shifted. This operation provided the registered reference signal used in the remaining LESM processing.

#### 2.2.2. Wavelet Denoising

Wavelet denoising was independently applied to the registered reference signal and the defect signal to reduce high-frequency fluctuations while retaining the oscillatory ultrasonic responses. In a J-level discrete wavelet decomposition, a signal can be represented by its approximation and detail components as

(3) $$x\left(n\right)=A_{J}\left(n\right)+\sum_{j=1}^{J}D_{j}(n)$$

where *A_J_*(*n*) denotes the approximation component at the decomposition level *J*, and *D_j_*(*n*) represents the detail component at the *j*-th decomposition level.

Wavelet denoising was performed using the MATLAB wdenoise function (MathWorks, Natick, MA, USA). An empirical Bayesian denoising method with a Cauchy prior, a posterior-median threshold rule, and a level-independent noise estimate was employed. The noise variance was estimated from the finest-scale wavelet coefficients. Thresholding was applied to the detail coefficients, after which the denoised signal was reconstructed while preserving the approximation coefficients.

The same wavelet family, decomposition level, and denoising options were applied independently to the reference and defect signals. Thus, the algorithmic settings were identical, while the noise characteristics and associated thresholds were estimated separately for each signal by wdenoise. Symlet wavelets from Sym3 to Sym6 and decomposition levels from 3 to 6 were evaluated using the DBR procedure described in Section 2.2.4. Sym5 with a decomposition level of 5 produced the highest evaluated DBR (22.496 dB) and was selected for the subsequent LESM processing.

For the auxiliary waveform-level comparison, the wavelet-denoised registered reference signal was subtracted from the wavelet-denoised defect signal. This waveform residual was used to evaluate the wavelet combinations and to illustrate the reduction of common structural responses. However, the final LESM signal was not generated by applying the envelope operation to this waveform residual. Instead, the Hilbert envelopes were independently obtained from the denoised reference and defect signals, as described below.

#### 2.2.3. Hilbert-Envelope Residual Weighting

The Hilbert transform was applied separately to the wavelet-denoised reference and defect signals. For either denoised signal, the corresponding analytic signal was defined as

(4) $$z\left(n\right)=x^{\left(w\right)}\left(n\right)+iH\left\{x^{\left(w\right)}\left(n\right)\right\},i=\sqrt{-1}$$

and its envelope was obtained as

(5) $$E\left(n\right)=\left|z(n)\right|=\sqrt{{\left[x^{\left(w\right)}\left(n\right)\right]}^{2}+{\left[H\left\{x^{\left(w\right)}\left(n\right)\right\}\right]}^{2}}$$

The reference and defect envelopes were smoothed using a centered moving-average window of 101 samples. The smoothed envelope can be written as

(6) $$\bar{E}\left(n\right)=\frac{1}{M}\sum_{k=-\frac{M-1}{2}}^{\frac{M-1}{2}}E\left(n+k\right),M=101$$

where *M* is the smoothing-window length. The selected odd-numbered window provides symmetric smoothing about each sample. Its approximate spatial span is 100*Δd*, where *Δd* is the distance increment per sample. At the signal boundaries, MATLAB movmean uses the available samples within the truncated window. The same window was used for the reference and defect envelopes.

The smoothed reference envelope was then subtracted from the smoothed defect envelope. Only positive differences were retained to emphasize locations where the defect-region envelope exceeded the sound-region envelope:

(7) $$R_{E}^{+}\left(n\right)=\max\left[{\bar{E}}_{d}\left(n\right)-{\bar{E}}_{r}\left(n\right),\;0\right]$$

This positive-residual operation suppresses common envelope components and excludes negative differences. Consequently, the present formulation does not explicitly use negative residuals that may arise from acoustic shadowing or a local reduction in transmitted amplitude.

The positive envelope residual was normalized by its maximum value to form the dimensionless weighting function

(8) $$W\left(n\right)=\frac{{R^{+}}_{E}\left(n\right)}{\underset{n}{\max}{{[R}^{+}}_{E}\left(n\right)]}$$

where 0 ≤ *W*(*n*) ≤ 1. Finally, the weighting function was multiplied pointwise by the wavelet-denoised defect signal to obtain the layer-echo-suppressed waveform:

(9) $$S_{\mathrm{LESM}}\left(n\right)=W\left(n\right)x_{d}^{\left(w\right)}\left(n\right)$$

The weighting function does not classify individual echoes directly. Instead, it is based on the premise that deterministic layer echoes and transducer ringing are similarly reproduced in the reference and defect signals. At depths dominated by these common structural responses, the smoothed reference and defect envelopes are similar, producing a positive envelope residual close to zero and consequently a low weighting value. These components are therefore attenuated. In contrast, a localized increase in the defect-signal envelope relative to the reference envelope produces a positive residual and a higher weighting value, allowing the corresponding portion of the denoised defect waveform to be retained. Because the weighting function is applied to the denoised defect signal rather than used as the final output, the oscillatory characteristics of the retained defect-related response are preserved. Thus, the distinction is based on localized envelope differences relative to the sound-region reference rather than on explicit classification of individual wave components.

#### 2.2.4. Wavelet-Parameter Evaluation and DBR Definition

The wavelet family and decomposition level were selected through a preliminary parameter evaluation using the defect-to-background ratio (DBR). For each of the 16 combinations of Sym3–Sym6 and decomposition levels 3–6, the independently denoised reference signal was subtracted from the independently denoised defect signal. The maximum absolute amplitude in the defect interval and the root-mean-square (RMS) amplitude in the background intervals were then calculated from this auxiliary waveform residual. DBR was defined as

$$\mathrm{DBR}=20\log_{10}\left(\frac{A_{\mathrm{defect}}}{A_{\mathrm{background},\mathrm{RMS}}}\right)$$

where a defect is the maximum absolute amplitude within the defect interval and background, RMS is the RMS amplitude of the selected background samples. The defect interval was defined as 48–58 mm to include the complete oscillatory response around the nominal 50-mm defect depth. The background was evaluated over 25–45 mm and 65–90 mm. These two intervals represent the surrounding repetitive layer-echo background while excluding the strong front-surface response, the defect-related waveform, and the back-wall reflection. The same intervals were applied to every wavelet combination and to the quantitative comparison of the processing stages.

The combination producing the highest DBR was adopted for the subsequent LESM processing. Because the parameters and performance metric were evaluated using the same representative defect signal, this optimization is treated as an in-sample proof-of-concept parameter selection rather than an independent validation of general detection performance.

## 3. Results and Discussion

### 3.1. Stepwise Signal Processing Using LESM

The proposed LESM described in Section 2.2 was applied to the ultrasonic A-scan signals acquired from the reference and defect regions. Prior to reference subtraction, cross-correlation analysis was performed to evaluate the temporal correspondence between the reference and defect signals. The optimal lag was determined to be 0 samples, with a maximum normalized cross-correlation coefficient of 0.9941. This high correlation indicates that the repetitive layer-echo patterns in the reference and defect signals were already well aligned within the selected registration region. Therefore, no additional temporal correction was required prior to reference subtraction.

To determine the wavelet parameters, Symlet wavelets from Sym3 to Sym6 at decomposition levels 3–6 were evaluated using the defect-to-background ratio (DBR). The resulting DBR values are summarized in Table 1.

The DBR values ranged from 22.4825 to 22.4958 dB, indicating relatively small differences among the evaluated combinations. Sym5 with a decomposition level of 5 produced the maximum DBR of 22.4958 dB and was therefore selected for the subsequent LESM processing.

**Figure 5:**
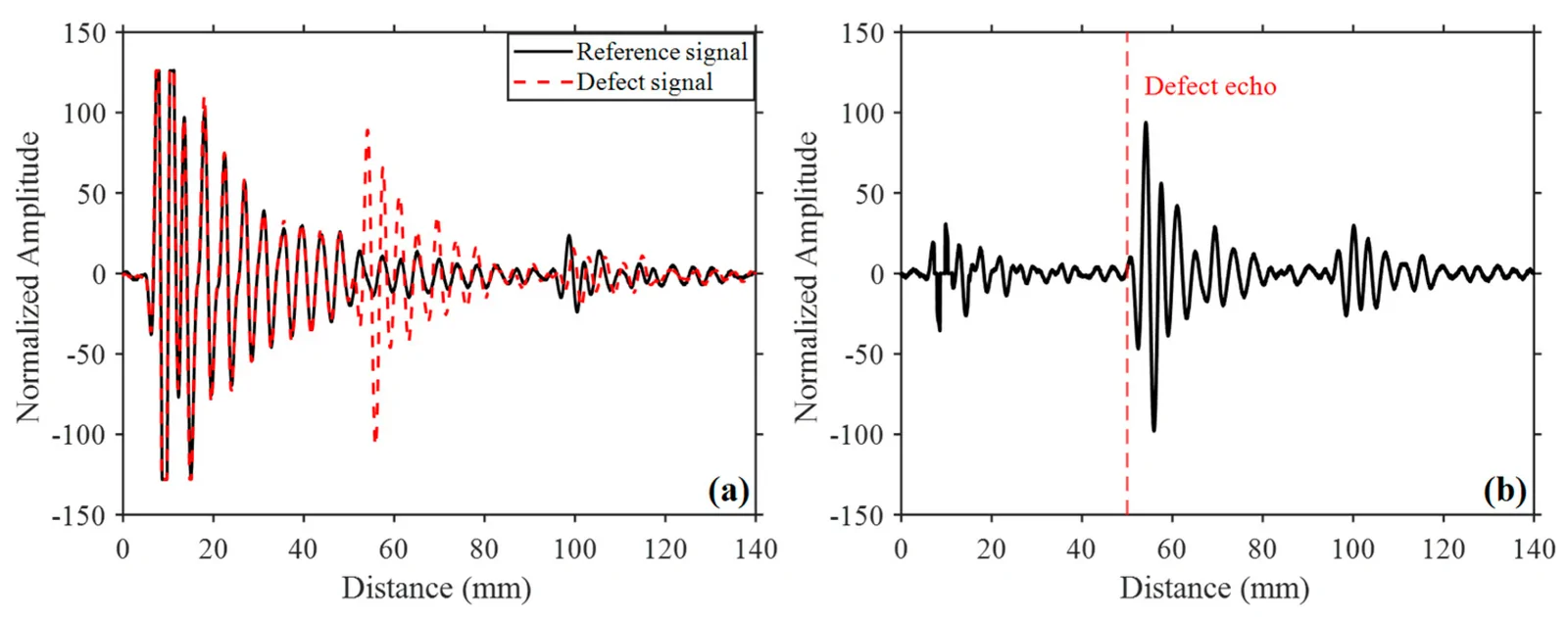
Wavelet denoising and reference subtraction results: (**a**) denoised reference and defect signals and (**b**) residual signal.

Figure 5a shows the reference and defect signals after wavelet denoising using the selected Sym5 wavelet with a decomposition level of 5. The repetitive layer-echo patterns remained highly similar between the two signals, while the defect-related response was preserved. Figure 5b presents the residual signal obtained by subtracting the wavelet-denoised reference signal from the defect signal. Owing to the high similarity of the common structural responses, reference subtraction substantially reduced the repetitive layer echoes while retaining the defect-related components. As a result, the relative prominence of the defect reflection at approximately 50 mm increased compared with that in the raw defect signal. Nevertheless, residual ringing components remained after reference subtraction. Hilbert-envelope analysis was therefore subsequently applied to further reduce the surrounding background components.

**Figure 6:**
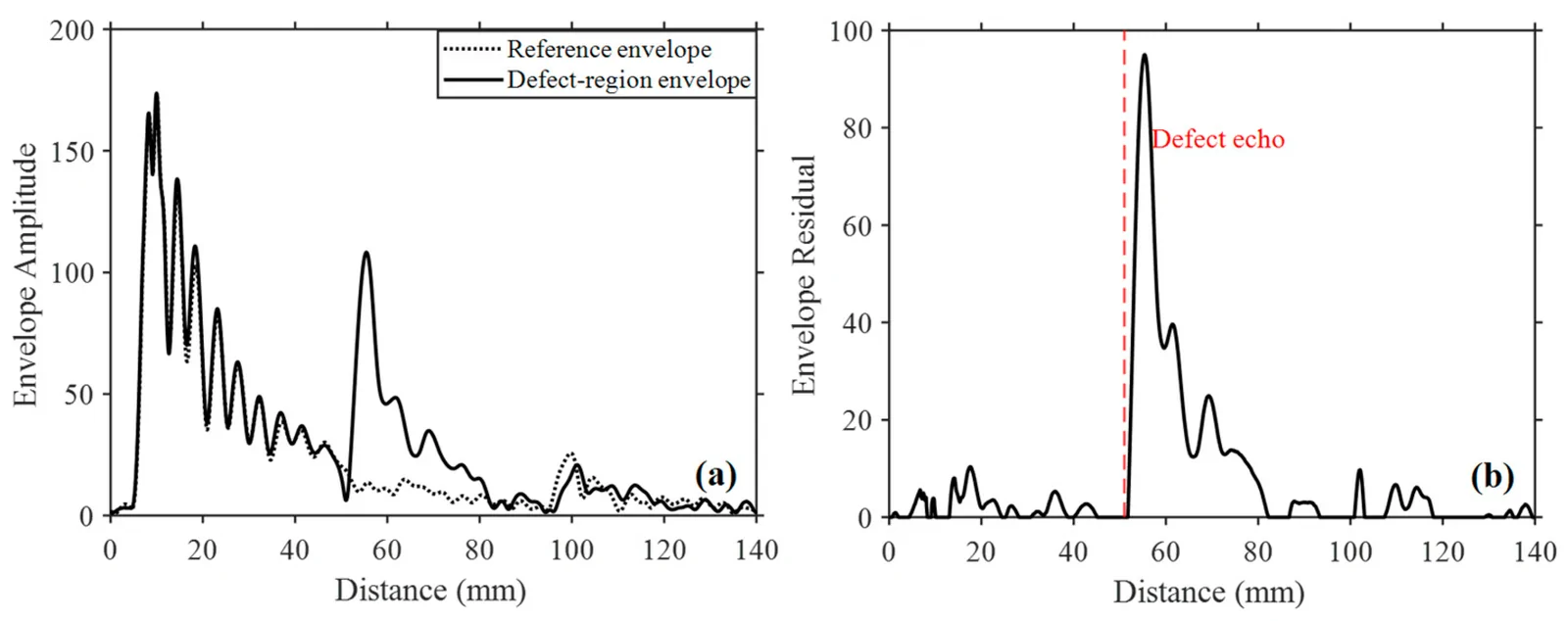
Hilbert-envelope analysis: (**a**) reference and defect envelopes and (**b**) positive envelope residual.

Figure 6 presents the Hilbert-envelope analysis of the wavelet-denoised reference and defect signals. As shown in Figure 6a, the reference and defect envelopes exhibit similar amplitude distributions in the common layer-echo regions, whereas a localized amplitude difference is observed near the defect location. In particular, the defect envelope shows an increase at approximately 50 mm, corresponding to the defect-related reflection. Figure 6b shows the positive Hilbert-envelope residual obtained from the difference between the defect and reference envelopes. The common layer-echo and residual ringing components are reduced, while the localized defect-related response is retained. Compared with the wavelet-based residual shown in Figure 5b, the positive envelope residual more clearly emphasizes the localized amplitude difference associated with the defect. The resulting envelope residual was subsequently normalized and used as a weighting function to generate the final LESM signal.

**Figure 7:**
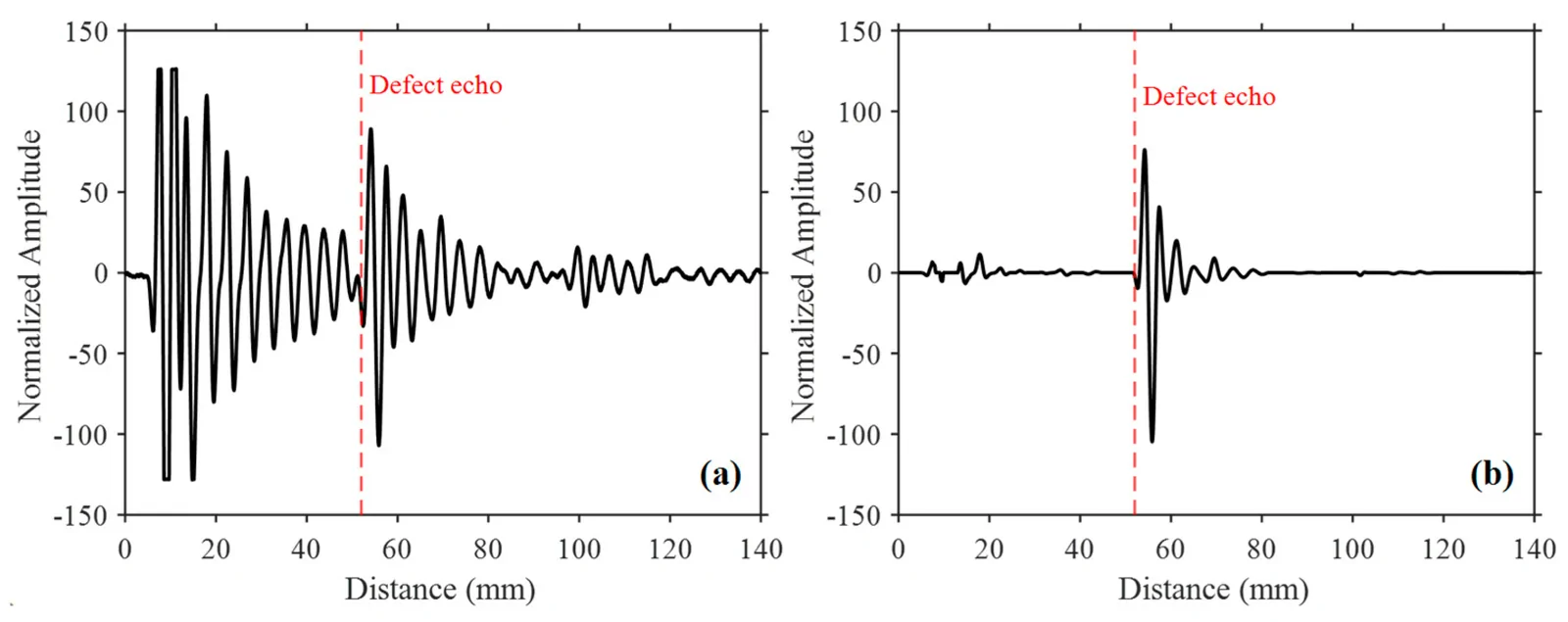
Comparison before and after LESM processing: (**a**) wavelet-denoised defect signal and (**b**) final LESM signal.

Figure 7 compares the defect signal before and after application of the proposed LESM.

As shown in Figure 7a, the wavelet-denoised defect signal still contains pronounced ringing and repetitive layer-echo components over a wide range of the inspection depth, although the defect reflection is observed at approximately 50 mm. To generate the final LESM signal, the positive Hilbert-envelope residual shown in Figure 6b was normalized and used as a weighting function. The weighting function was then multiplied by the wavelet-denoised defect signal, thereby selectively retaining signal components associated with the defect response while suppressing regions dominated by common structural responses. As shown in Figure 7b, the repetitive layer echoes and residual ringing components are substantially reduced, while the defect-related waveform is retained. Consequently, the final LESM signal exhibits improved defect-to-background separation compared with the wavelet-denoised defect signal.

### 3.2. Quantitative Performance Comparison

**Figure 8:**
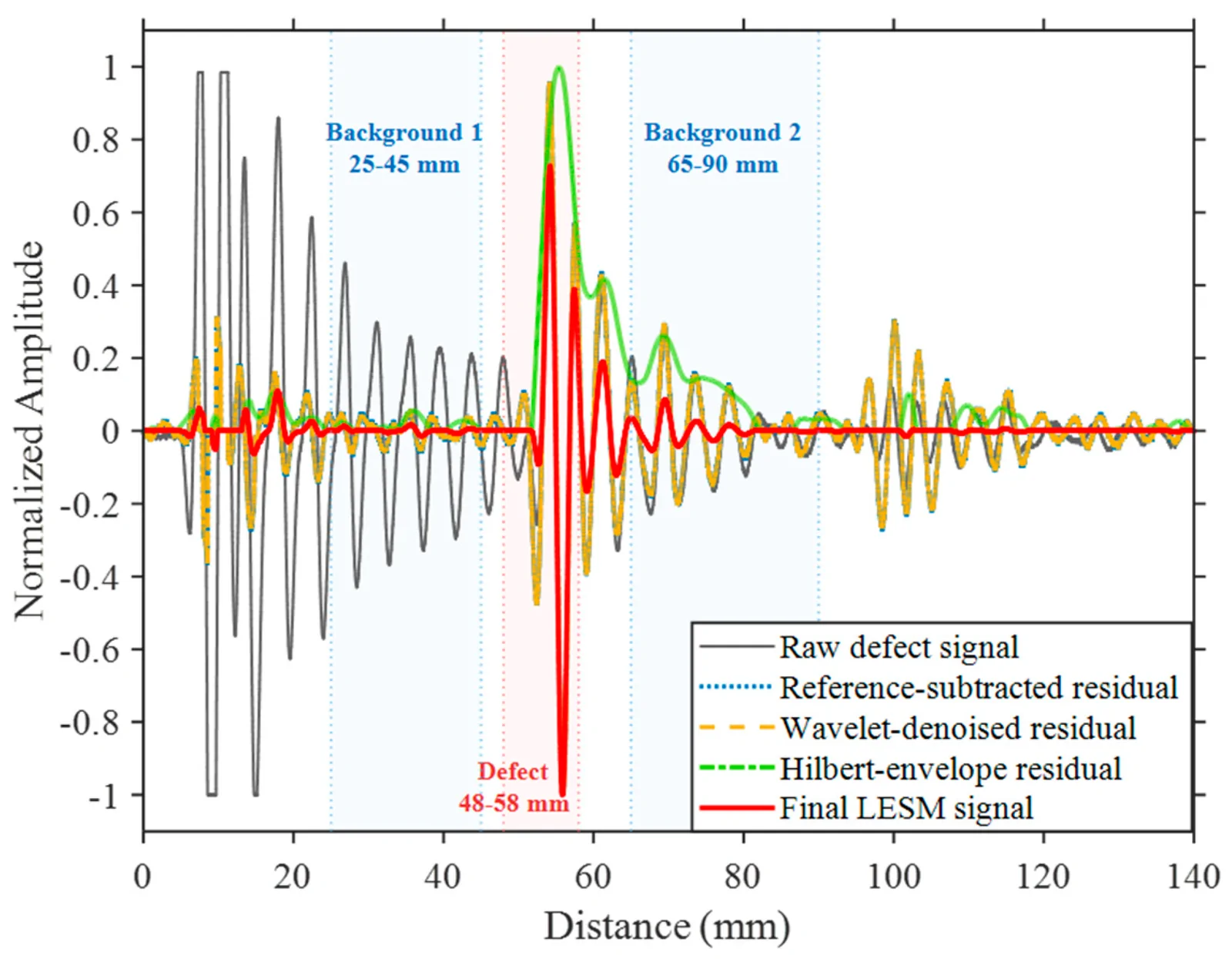
Normalized signal comparison at different LESM stages with DBR evaluation intervals.

Figure 8 compares the normalized ultrasonic signals obtained at different stages of the proposed LESM. For direct comparison of the relative background responses, each signal was normalized by its maximum absolute amplitude. The raw defect signal exhibits pronounced ringing and repetitive layer echoes over a wide range of the inspection depth, resulting in a relatively high background level around the identifiable defect reflection. After reference subtraction, the common structural responses are substantially reduced, thereby increasing the relative prominence of the defect reflection near 50 mm. Wavelet denoising further reduces small background fluctuations while retaining the principal defect-related waveform.

Following Hilbert-envelope analysis, the amplitude difference associated with the defect becomes more localized, while the remaining layer-echo and ringing components are further reduced. The final LESM signal exhibits the lowest relative background response among the evaluated processing stages while retaining the dominant defect-related waveform. In particular, the ringing components observed before the defect location and the residual structural responses after the defect are markedly reduced. These results demonstrate the progressive suppression of common structural responses through the sequential processing stages of the proposed LESM.

To quantitatively compare defect-to-background separation at successive processing stages, the DBR was calculated using the definition and evaluation intervals specified in Section 2.2.4. The same defect and background intervals were consistently applied to all signals to enable a direct comparison among the processing stages.

**Figure 9:**
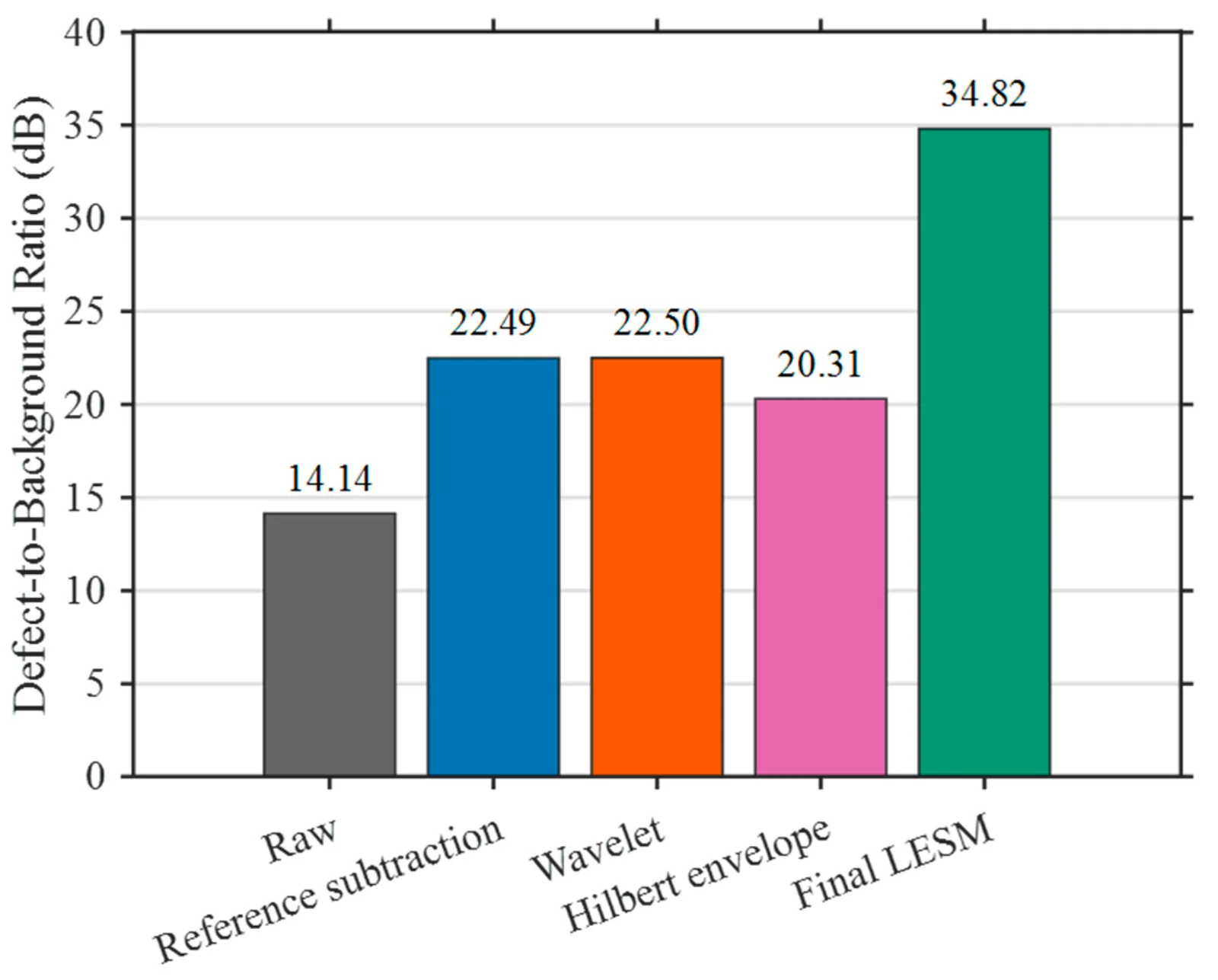
Defect-to-background ratio at each LESM processing stage.

Figure 9 presents the DBR calculated at each stage of the proposed LESM. The raw defect signal exhibited a DBR of 14.14 dB because of the pronounced ringing and repetitive layer echoes surrounding the defect response. After reference subtraction, the DBR increased to 22.49 dB, corresponding to an improvement of approximately 8.35 dB. This increase indicates that subtraction of the reference signal effectively reduced the common structural responses while retaining the defect-related reflection.

Wavelet denoising resulted in a DBR of 22.50 dB, which was nearly identical to that obtained after reference subtraction. This result indicates that wavelet denoising primarily reduces high-frequency fluctuations and has a limited direct effect on the repetitive structural echoes. Accordingly, wavelet denoising was retained as a preprocessing step before Hilbert-envelope calculation rather than as the primary layer-echo suppression stage. Following Hilbert-envelope processing, the DBR was 20.31 dB. Although the envelope residual facilitated localization of the defect-related response, the DBR alone did not increase at this intermediate stage because the envelope transformation changes the amplitude distribution of both the defect and background components.

In contrast, the final LESM signal exhibited the highest DBR of 34.82 dB, representing improvements of 20.68 dB over the raw defect signal and 12.33 dB over direct reference subtraction. The DBR after wavelet denoising was 22.50 dB and was nearly identical to the value of 22.49 dB obtained after reference subtraction. The substantial additional improvement was therefore achieved only after the positive Hilbert-envelope residual was used as a weighting function for the denoised defect signal. Unlike direct subtraction, which uses the signed waveform difference as the output, the proposed weighting stage attenuates depth intervals dominated by common structural responses while retaining the oscillatory defect-related waveform in intervals where a localized envelope excess is present. This comparison quantitatively demonstrates the contribution of the reference-derived envelope-residual weighting stage under the evaluated condition. Because the main stepwise comparison and parameter selection were performed using Acquisition 1, the observed improvement should be interpreted as proof-of-concept evidence rather than as independent validation of general detection performance.

The performance of LESM is also expected to depend on the input signal-to-noise ratio (SNR) because the method compares two independently acquired A-scan signals. Under relatively high-SNR conditions, deterministic layer-echo patterns dominate the reference and defect signals, allowing cross-correlation to identify their temporal correspondence and the envelope residual to emphasize the localized amplitude difference associated with the defect. As the input SNR decreases, random noise may lower and broaden the cross-correlation peak, increasing uncertainty in signal registration. Although wavelet denoising can reduce stochastic high-frequency fluctuations, noise components overlapping the frequency band of the ultrasonic response may remain, and subtraction of independently acquired signals may further increase the residual noise. Lower input SNR may also produce random positive differences between the Hilbert envelopes of the reference and defect signals. Because these residuals are used to construct the LESM weighting function, noise-induced weighting outside the defect region may increase the final background level and reduce the resulting DBR improvement. The normalized cross-correlation coefficient of 0.9941 obtained in the present experiment indicates strong waveform similarity for the evaluated signal pair but does not establish robustness over a range of input SNRs. Therefore, the limiting SNR of LESM cannot be determined from the present fixed-condition measurements, and a controlled study involving different input SNRs and repeated noise realizations is required to establish its quantitative operating range.

A limited repeat-acquisition assessment was additionally performed to examine the sensitivity of LESM to manual transducer removal and repositioning. After the first measurement, the transducer was completely removed and manually repositioned at the same nominal defect location, and a second A-scan was acquired under the same instrument settings. The second acquisition was processed using the same reference signal and fixed LESM parameters, without re-optimization. The results are summarized in Table 2.

The normalized correlation coefficients with the reference signal were 0.9941 and 0.9947 for Acquisitions 1 and 2, respectively, with an estimated alignment lag of zero samples in both cases. The raw-signal DBRs were 14.14 and 13.22 dB, whereas the corresponding final LESM DBRs were 34.82 and 35.24 dB, representing improvements of 20.68 and 22.02 dB. The detected peak positions differed by only 0.022 mm, and the defect-peak retention values relative to the corresponding wavelet-denoised defect signals were 97.74% and 95.91%, respectively. These results indicate that the suppression performance was maintained after a single independent manual re-contact under the examined condition. Nevertheless, because only two acquisitions from one defect location were available, this comparison should be regarded as a limited repeat-acquisition assessment rather than a statistical validation of repeatability.

Although the transducer was positioned approximately normal to the specimen surface, the measured A-scans may contain scattered and mode-converted wave components because pultruded CFRP is anisotropic and heterogeneous. Minor variations in probe angle and acoustic coupling during manual measurements may alter these components and contribute to the residual oscillations. LESM does not explicitly distinguish among individual propagation modes. Mode-converted components that are similarly present in both the reference and defect signals are treated as part of the common structural response and attenuated through reference-based processing. In contrast, components caused by local anisotropy, probe tilt, or coupling differences may not match the reference signal and may therefore remain in the residual background. Further studies under controlled probe-angle and coupling conditions are required to quantify these effects.

The intended application of the present method is the manufacturing-stage inspection of pultruded CFRP components before their integration into a wind turbine blade, rather than the in-service inspection of a completed blade structure. At this stage, measurements can be performed within production sections having the same nominal thickness and material architecture, allowing a representative reference signal to be acquired from a verified sound region of the same component or from a defect-free component manufactured under the same conditions. Therefore, the acquisition of a structurally comparable reference signal is considered feasible for the intended application. If LESM is subsequently extended to completed components with tapered or variable-thickness geometries, a single reference signal may no longer be sufficient. Such applications would require locally acquired or thickness-matched reference signals and additional validation.

## 4. Conclusions

This study proposed a reference-based Layer Echo Suppression Method (LESM) to improve defect-to-background separation in the ultrasonic evaluation of thick pultruded CFRP composites. The methodological contribution of LESM lies in its reference-derived envelope-residual weighting architecture. Rather than using direct reference subtraction as the final output, LESM derives a non-negative weighting function from the positive difference between the smoothed Hilbert envelopes of the denoised defect and reference signals. This weighting function is then applied to the denoised defect signal to attenuate common layer echoes and ringing while retaining the oscillatory defect-related waveform.

Cross-correlation analysis confirmed a high temporal correspondence between the reference and defect signals, with a normalized cross-correlation coefficient of 0.9941, supporting the use of the sound-region signal as a structural reference. Wavelet parameters were evaluated based on the defect-to-background ratio (DBR), and Sym5 with a decomposition level of 5 was selected for subsequent processing. Reference subtraction reduced the common layer-echo components, while Hilbert-envelope analysis localized the amplitude difference associated with the defect. The resulting positive envelope residual was subsequently used as a weighting function to reduce the remaining structural responses.

The final LESM signal exhibited a DBR of 34.82 dB, compared with 14.14 dB for the raw defect signal and 22.49 dB after direct reference subtraction. These values correspond to improvements of 20.68 dB over the raw signal and 12.33 dB over direct subtraction. The additional improvement obtained through envelope-residual weighting indicates that the complete LESM procedure provides greater defect-to-background separation than waveform-level reference subtraction alone under the evaluated condition. The defect-related waveform at approximately 50 mm was retained, while the surrounding layer echoes and ringing components were substantially reduced.

Because LESM is implemented as a post-processing method, it can be applied to conventional pulse-echo A-scan measurements without additional ultrasonic hardware. Its practical application, however, requires a representative sound-region signal having a structural response comparable to that of the inspection region. Because LESM is implemented as a post-processing method, it can be applied to conventional pulse-echo A-scan measurements without additional ultrasonic hardware. Its practical application, however, requires a representative sound-region signal having a structural response comparable to that of the inspection region. The effectiveness of LESM therefore depends on sufficient similarity between the common structural responses of the reference and defect signals, although the present results do not establish a universal minimum correlation coefficient. Cross-correlation can be used to assess reference suitability, and a locally or structurally matched reference should be selected when the correlation is low. The present proof-of-concept is primarily intended for manufacturing-stage inspection of pultruded CFRP components having locally uniform thickness. Extension to completed tapered or variable-thickness structures would require locally acquired or thickness-matched reference signals and further validation. The present proof-of-concept is primarily intended for manufacturing-stage inspection of pultruded CFRP components having locally uniform thickness. Extension to completed tapered or variable-thickness structures would require locally acquired or thickness-matched reference signals and further validation.

In addition, the present study considered a single planar PTFE insert at a fixed depth in a uniform 100-mm-thick specimen, and the positive-residual weighting strategy does not incorporate negative residuals associated with acoustic shadowing. The reported DBR improvement should therefore be regarded as a proof-of-concept result rather than a comprehensive assessment of probability of detection or defect-sizing accuracy.

A limited repeat-acquisition comparison showed comparable LESM performance after the transducer was removed and manually repositioned at the same nominal defect location. However, statistical repeatability remains to be established through a larger number of independent measurements covering different coupling conditions, operators, and spatial locations. Further studies involving defects with different depths, sizes, and irregular morphologies are required to establish the broader applicability of LESM.

## Figures and Tables

**Table 1:** DBR values (dB) for the evaluated Symlet wavelets and decomposition levels.

Wavelet	Level 3	Level 4	Level 5	Level 6
Sym3	22.4862	22.4887	22.4895	22.4896
Sym4	22.4874	22.4886	22.4884	22.4881
Sym5	22.4825	22.4919	22.4958	22.4954
Sym6	22.4867	22.4864	22.4863	22.4859

**Table 2:** Comparison of independently acquired defect-region A-scans processed using identical LESM parameters.

Acquisition	Reference Correlation	Raw DBR (dB)	LESM DBR (dB)	DBR Gain (dB)	Peak Retention (%)
1	0.9941	14.14	34.82	20.68	97.74
2	0.9947	13.22	35.24	22.02	95.91

Note: Reference correlation denotes the maximum normalized cross-correlation coefficient with the sound-region reference signal. Peak retention was calculated as the maximum absolute amplitude of the final LESM signal divided by that of the corresponding wavelet-denoised defect signal within the predefined defect interval.

## Data Availability

The data supporting the findings of this study are available from the corresponding author upon reasonable request.
